# Hospital and Institutional News

**Published:** 1913-11-22

**Authors:** 


### HOSPITAL AND INSTITUTIONAL NEWS.

### UNISON IN SPECIAL CHRISTMAS APPEALS:
Our Christmas Prizes.

The Hospital this year, as usual, will publish a Special Appeal Number, exhibiting what the spirit of personal service achieves for those who give and those who receive. In order to bring home to the public what is the spirit of personal service within the institutions themselves, we offer a prize of two guineas each for the best illustrated account or one guinea for the best account without illustrations of "How to Spend Christmas in a Hospital," from the five points of view of the Resident Medical Staff, the Secretariat, Matron, Sister, and Nurse. Every type of institution is included, and *in addition* a special prize of three guineas will be awarded to the best essay (with illustrations) of the five points of view. Any writer therefore has a chance of winning five guineas, and the essays will reveal how few or how many institutional workers there are in the country alive to the spirit of personal service in themselves, and to a conscious understanding of it as the driving force in institutional life. Prize essays must reach us not later than December 8. They should be written plainly (preferably typed) on one side of the paper only, contain not more than 1,000 words, and the Editor's decision and awards are to be final and unquestioned. The prize essays will be published in the Christmas Number.

### TWO NEW MENTAL COMMISSIONERS.

On the recommendation of the Home Secretary the King has appointed Mr. W. H. Dickinson, M.P., and Mrs. Pinsent to be Commissioners, unpaid, under the Mental Deficiency Act.

### THE FUTURE OF CARDIFF MEDICAL SCHOOL.

Hardly had we gone to press last week when a special general meeting of the governors of King Edward VII.'s Hospital, Cardiff, was held under the presidency of Major-General Lee, who moved that the rules be altered to ensure that no person should be appointed to the staff of the hospital as physician or surgeon unless he held the respective qualification of F. or M.R.C.P. London, or F.R.C.S. England. A clause was inserted in the proposal exempting from such regulation the assistant physicians and surgeons at present on the staff. In moving the adoption of the proposed rule General Lee pointed out the desirability of having a medical school associated with the hospital and the best way of making such a school effective. Professor Hepburn, Dean of the Medical Faculty of the University College of South Wales and Monmouthshire, who seconded the resolution, contended that the request for special diplomas by electing committees could not possibly be regarded as a slight upon the holder of a degree or upon the University which conferred it. In the University of London there were six different types of M.D. Colonel Bruce Vaughan, who followed, explained that opportunities for teaching stimulated a hospital staff to an enhanced standard of efficiency, while the prosecution of research redounded also to the advantage of medical science. Exception was taken to the proposed rule by Dr. Tenison Collins and others, on the ground that the restrictions suggested might have the effect of excluding brilliant men who did not possess the specified qualifications. Although the meeting ultimately passed an adverse verdict on the resolution by a majority of three, the impression left from the general discussion was that both parties, however much they might differ in details, were united in their desire to ensure that in future appointments to the staff only men of the highest qualifications and attainments should be elected. The future is in the hands of the election committee, the constitution of which is certainly efficient.

### NEW ASSOCIATION OF MENTAL MEDICAL OFFICERS.

At Birmingham on Tuesday a meeting of Midland assistant medical officers in mental hospitals was held, at which Dr. Stuart, of Northampton, discussed the grievances and disabilities under which the junior members of the mental hospital service continue to labour. Dr. Stuart went over the well-known stony ground of small prospects and small salaries. After many years' work, he asserted, the assistant medical officer's salary is often only a beggarly £50 a year more than that offered in vain as an inducement to juniors to enter the service. The marriage difficulty—its prohibition or virtual prohibition in the absence of married quarters—was also described, and the speaker asserted that promotion depended on local influence. It was finally resolved to form an association of assistant medical officers of public mental hospitals provisionally to represent the Midlands. We have gone over this ground so often that we can sympathise with this latest endeavour to remedy the despair that so often eats into mental officers' lives, and shall watch with interest the new association's growth and progress.

### THE NEW TRANMERE INFIRMARY.

Practically complete, the new infirmary associated with Tranmere Workhouse has been inspected by the works committee. The architect, Mr. Edmund Kirby, Col. G. F. Allender, chairman of the works committee, and the Guardians visited the institution, which has cost just under £60,000. Mr. Augustin Quinn, chairman of the Board, explained that twelve years ago Dr. Fuller and Mr. Dansey, on behalf of the Local Government Board, complained of the old buildings. Under pressure from this Department the reconstruction was planned. Mr. Kirby described the old buildings, where, he said, he had found only one bath, which was in the basement under the dining-hall, and used mainly for washing potatoes. The bathroom in the isolation block was used as a servants' bedroom. The old hospital contained 132 beds, the new 322, and the cost per head was £98, a commendably low figure in the official view. By favour of the Local Government Board, which first condemned the old site as impossible, the scheme has been carried out piecemeal, but even to-day the quarters of the infirmary medical superintendent and offices and a maternity ward, with infants' accommodation, still remain to be completed. The inspection covered two new ward blocks and an administration block. The former include surgical, acute medical, general medical, contagious diseases, chronic, and blind wards. Dr. Stansfield, medical officer to the workhouse, whose connection with the Board began in 1897, was appointed to his present post eight years ago. Since 1908 the new admissions have risen from 1,200 to 2,000. He declared that the new institution would compare favourably with any in the neighbourhood.

### CHARGES AGAINST SHEFFIELD CITY HOSPITALS.

In view of the serious charges that have been made against Dr. Egerton H. Williams, medical superintendent of the city hospitals, and a sister at Lodge Moor Hospital, the City Hospitals Committee have issued a public statement from which we take the following:—

The Town Clerk reported that summonses had been issued under Section 12 of the Children Act, 1908, by Herbert Cadman, of 70 Rushdale Avenue, Meersbrook, against Dr. Egerton H. Williams, medical superintendent of the city hospitals, and Miss Amelia Scott, sister-in-charge of Ward 4 South at Lodge Moor Hospital, alleging against Dr. Williams on various days between June 20 and July 24, 1913, the neglect of Wilfred Cadman, a child under three years of age, and alleging against Sister Scott assault, ill-treatment . . . The child referred to died in the hospital on July 24 last, after a second attack of scarlet fever, which developed during the convalescent stage of the first attack. The summonses were based upon the practice which obtains at Lodge Moor—a practice which is accepted by medical men as proper and is followed in similar institutions throughout the country—of soaking the hands and feet of all scarlet fever patients in warm water as soon as they are convalescent and able to be up in clothes in order to expedite the peeling of the skin which accompanies an attack of scarlet fever. It was alleged by the prosecution that the staff at the hospital failed to renew sufficiently often the warm water in which the hands and feet of the patient were soaked, and by allowing the water to become cold they endangered the health of the patients, and in this particular instance caused the death of the child.

"No evidence whatever," the report proceeds, "was adduced by the prosecution in support of the serious allegations made . . . and at the conclusion of the informant's case the Stipendiary Magistrate . . . dismissed the summons against Sister Scott," whereupon that against Dr. Williams was withdrawn. The committee have expressed their gratitude to the medical superintendent and the nursing staff, and their belief that the charges were "altogether unwarranted." The Town Clerk had been instructed to defend the proceedings, to retain counsel, and call evidence at the expense of the Corporation. Dr. Williams' victory could hardly, it seems, be more complete, and we congratulate him and the city hospitals upon this authoritative public tribute to their work and the spirit in which it is conducted.

### THE EXTENSION OF MANSFIELD HOSPITAL.

Three times since its opening in 1890 has the Mansfield and Mansfield Woodhouse District Hospital been enlarged. The third occasion was formally inaugurated when the Duke of Portland and a representative group of Freemasons were present at the laying of the foundation-stone of the new wing which forms the local memorial to King Edward. At a cost of £11,600, of which rather more than half has been subscribed, a three-storey building has been erected containing in all fifty-three beds, distributed between three large and three small wards, while nurses' rooms and the usual completions of the ward units have been included. Mr. F. N. Ellis, the hospital's president, has announced new subscriptions, and Mr. H. E. Hollins, chairman of the monthly board, summarised its history from the pre-colliery days of 1867 to the present time, when the coal-pits provide patients in large numbers. He urged that a new isolation ward should be built, and foreshadowed further improvement in three years' time, the date of the hospital's jubilee. Certainly no hospital in a mining district should be poorly supported. It is the friend in emergencies, the ever-ready aid for the ever-possible accident, and subscriptions to it are the cheapest form of insurance that colliery companies can make; one, too, which gives them a kudos which the size of their subscriptions often hardly deserves.

### NEW METHODS IN MENTAL HOSPITALS.

Dr. W. E. Jones, Inspector-General of Insane Asylums in Victoria, has presented his annual report to Parliament before leaving for a brief holiday. In Europe he represented his State at several medical congresses, and interested the International Medical Congress at the Albert Hall in a paper on the "Newer Methods of Lunacy Administration" which have been adopted throughout Australia. Now Dr. Jones is on his return, bringing with him the impression that there is cause for alarm at the increase of insanity throughout the world during recent years. The report mentioned—for the year 1912—gives the number under treatment as 5,579, an increase of 130 over 1911. Ratio of insane to population, 1 in 246.5. Admitted during year, 806, compared with 817 in 1911 and 802 in 1910. Insanity is stated to have increased among men and decreased among women. Of the new cases dealt with, 152 were attributed to heredity, 76 to venereal diseases, 114 to alcoholism, and 78 to congenital defects. Eighty-three patients were under treatment in private asylums. Death-rate for 1912, 7.82, which was lower than in 1911; 56 of the deaths were from general paralysis.

### THE CHIROPODY HOSPITAL.

It is always wise to keep an eye upon new professions. Institutional workers will have therefore noticed the opening last week of an institution at 1 Silver Street, Bloomsbury, where patients who cannot afford the ordinary fees for treatment will be freely attended to by honorary members of the

National Society of Chiropodists, which was formed and incorporated some twelve months ago. The institution, or out-patient department, consists of two floors, in which are two clinics with full equipment. The hours of attendance are from 7.30 to 9.30 on Mondays and Thursdays, when men and women patients will be attended to respectively. During the day the staff is engaged in private practice. We are glad that chiropody is now being taken seriously. Only the other day a medical man told us that he had come up to London and sacrificed one of his toes rather than continue with the discomfort which a corn had caused him. He "confidently recommended" this course to others in a case of like pain, but the drastic method he employed shows how unscientifically chiropodic cases have been treated hitherto. The special study of it should gain in prestige and importance from the new institution.

### THE MEDICAL INSPECTION OF FACTORIES.

Mr. John C. Bridge, F.R.C.S., D.P.H., has been appointed by Mr. McKenna medical inspector of factories. Previous to this appointment Mr. Bridge has been medical officer of health for the county of Brecon. The successful reformer is the man who is instant in season and out of season in pressing his aims. We therefore make no apology for reminding our readers of the recent exposure of the unhealthiness of most offices, and for the urgent need of authorising medical inspectors of factories to inspect and report on the conditions of the offices as well as of the workshops through which they pass. Secondly, we may repeat the urgency that exists for adding to the number of medical inspectors under the Poor Law, a need which Dr. Deane Sweeting's death enabled us to emphasise on behalf of all infirmary medical superintendents.

### THE HOME OFFICE AND DR. DIMOCK.

The resumed inquest held last week on the late Dr. Horace Dimock, the panel doctor of Wisbech, which has led to demonstrations on the part of the public in the district in the idea that he was persecuted by the non-panel medical men, has been adjourned to December 3, so that Dr. Willcox, Home Office expert, may complete his examination. Dr. G. Martin Hall, the Coroner for the Isle of Ely, warned the jury in his address that they were not concerned with the allegations of persecution, but with the cause of death, which the forthcoming Home Office opinion will largely determine. He added that the policeman had tried at three different places to get an analysis of the deceased's viscera, and had been refused in each case. The analysis was needed because the post-mortem revealed no cause of death. The Coroner added in conclusion that he thought other aspects of the case should be taken up by the medical societies, who ought to get a special jury to inquire into the circumstances. Almost simultaneously the Isle of Ely local medical committee agreed that in view of the accusations of persecution the National Health Commission be asked to make a judicial inquiry into the circumstances of Dr. Dimock's death. A resolution was also passed expressing entire confidence in the committee's Chairman, Dr. H. F. Meacock, and his medical colleagues in Wisbech.

### PROPOSED AMERICAN WARD IN LONDON HOSPITAL.

We are sorry to see from the facts sent us by Mr. J. R. Coughlan, the secretary of this scheme, that it seems sadly to lag. The patronage list is described as "the largest and most influential ever organised for any American object in London," and the names quoted would seem to justify the claim. When, however, we turn to the list of subscriptions received and promised, the most striking features about it are first of all the absence of the names of the majority of the patrons from the list, and next the smallness of the individual sums contributed. We called attention to this scheme in The Hospital of October 18, p. 57, and the fact that the sum so far promised is not even enough to endow a single bed, much less a ward, indicates that those responsible should hustle or the scheme may necessarily fail from inanition. From the British point of view it would be very unfair to exploit a great voluntary hospital like the London and not secure the spontaneous completion of the scheme without delay.

### MR. AUSTEN CHAMBERLAIN'S APPEAL CLOSED.

At a meeting held at the London Chamber of Commerce last week the Fund raised by the City Sub-Committee on behalf of the London School of Tropical Medicine was formally closed. The report presented by Mr. P. Michelli, C.M.G., the secretary, showed a total of £71,444. No bad fraction, as Mr. Chamberlain pointed out, of the £100,000 for which he had appealed. He pointed out that only £15,000 had been put into bricks and mortar, and that £39,000 would be invested for the benefit of the general funds of the School. The medical staff and the School had co-operated to arrange a scheme for the benefit of those returned from the Tropics who were unable to pay for the special treatment they required. The "Chamberlain" Ward has been endowed for them.

### THE SUPERINTENDENTSHIP AT DARENTH.

The appointment of Dr. A. Rotherham, as already announced in The Hospital, to be a Commissioner (paid) under the Mental Deficiency Act has led the Metropolitan Asylums Board to consider the salary and successor to the post of medical superintendent of Darenth Industrial Colony, from which Dr. Rotherham has been transferred. At last Saturday's meeting of the Board both subjects were discussed. The proposal of the Finance Committee was that the salary attached to the superintendentship of Darenth Colony be £700, rising by four annual increments of £50 to £900, with emoluments. The Committee, it was said, proposed to appoint one of their present medical superintendents, whereupon the advisability of advertising the vacancy was strongly urged by several speakers, who emphasised the need for a first-rate man in view of the importance which would belong to the post under the Mental Deficiency Act. On this head it was also urged that a higher salary than that proposed would be required to attract the best class of candidates.

### CENTRALISING THE CARE OF MENTAL DEFECTIVES.

Representatives of numerous societies concerned in the care and control of the feeble-minded met at Denison House last Saturday, with Mr. Ernest Pollock, K.C., M.P., in the chair. He emphasised the need for adopting a combined method in which all the existing voluntary agencies could join to carry out the provisions of the Mental Deficiency Act. The voluntary organisations, he said, were essential to the local authorities, which, unaided, could not provide for the guardianship of persons under supervision. The Government, he understood, intended to make a grant to voluntary associations. Under the shadow of Denison House the spirit of centralisation of organisations inevitably grows, and a resolution was passed forming a central association for the care of mental defectives, which will consist chiefly of representatives of county and county borough associations, of subscribers and co-opted experts of institutions and associations concerned in the work, who will form a central council. It is intended thus to provide a representative body with which the Government can confer or communicate, which can distribute Government and other funds and organise the work of caring for those defectives who are not supervised in institutions. The formation of this council should mark a useful step forward. For whatever else the Mental Deficiency Act may do, it will certainly involve, if carried out with any thoroughness, an enormous and largely unappreciated expenditure, to lessen which the Government will be only too glad by getting into close touch with the voluntary associations. If only they all join by sending representatives to the central association an organ will have been created with which the Government can treat.

### THE CRYING OF HOSPITAL CHILDREN.

An influential committee of Ealing public men, including the Mayor, Councillor J. G. Eden, J.P., Mr. Herbert Nield, K.C., M.P., Drs. C. A. Patten and Fenton, have recently decided to appeal for funds to meet the erection of a children's ward at the above-mentioned hospital to form a perpetual memorial to the late Mr. Charles Jones, for over fifty years borough surveyor and engineer of Ealing, who died a few months ago. The ward will be known as the "Charles Jones Memorial Ward." The cost is estimated at about £1,500. Alderman Box stated that the house committee of the hospital had approved a plan of such a ward long before the proposition in the present case was thought of. The crying children in adults' wards was disconcerting to the older patients, and the upkeep of the new addition would cost very little more, while its provision would give both the children and the adult patients a much better chance of quick recovery. The proposal for the new ward was unanimously adopted, and energetic steps are being taken in the matter. There can be little doubt of the need for keeping children separate from adults in hospital wards. Crying is sometimes inevitable, even though its presence or absence is one of the best tests we know of the efficiency of hospital, especially children's hospital, administration and nursing.

### LIVERPOOL'S NEW HEALTH COMMITTEE.

The first meeting of the newly formed Health Committee of the Liverpool Corporation was held last week, with Mr. H. R. Rathbone, the Lord Mayor, in the chair. A new chairman, in place of Alderman Thomas Menlove, was appointed in the person of Alderman Muirhead, who has previously acted as chairman of the sanitary sub-committee. In an interesting speech the new Chairman stated that they had several important matters before them. Queen's Drive, not only a new thoroughfare, but a health lung, would be in part completed during the year. The bringing in of the outer districts would require careful attention. Mr. A. L. R. Rathbone was appointed deputy-chairman. Dr. Utting has been re-elected chairman of the Port Sanitary and Hospitals Committee. The increasingly wide recognition of the importance of Public Health work, and of the committees to which it is entrusted, make the *personnel* responsible for carrying it on of interest and importance to the community to a degree that certainly was not foreseen a few years ago.

### LONDON'S AMBULANCE AND THE COUNTY COUNCIL.

As our readers will have expected from the Note in The Hospital on the subject last week, the meeting of the London County Council on Tuesday did not advance the chances of London having an adequate ambulance service in the near future. Owing to the action of the Whips, the question resolved itself into a party one, and the Progressive proposal for the appointment of a special committee to consider the best methods by which the Council itself can organise an ambulance system was defeated by sixty-six to forty-four. Mr. Percy Harris, however, deserves thanks for having moved this amendment, but it was eventually decided to invite the Metropolitan Asylums Board to prepare a scheme in co-operation with the Council for the formation of an official ambulance service for London. Mr. W. H. Ecroyd, a member of the Metropolitan Asylums Board, said if the Board started the work it would, like the Council, have to create a new organisation. The Council ought not to shirk its duties; but what opinion can be formed of a public body which converts a question of street ambulances into one for its party Whips to decide?

### SIR GEORGE NEWMAN ON THE OPEN-AIR SCHOOL.

Sir George Newman, M.D., Chief Medical Officer of the Board of Education, delivered the second of his series of three lectures on "The Place of Open Air in Preventive Medicine" at the University of London last week. Sir George Newman said that the ventilation of the ordinary elementary public schools of the country was at present very much open to criticism; the amount of carbonic-acid gas was in some cases double the quantity which ought to have been permitted, and he said these conditions were representative. Consequently what they were aiming at in the open-air process was precisely opposite to what they were getting in the ordinary schools of the country. In an open-air school a child would get cheap and nourishing food given to it at regular intervals and under suitable conditions. Scarcely enough importance had been attached to the value of proper rest in cases of surgical tuberculosis and he showed pictures on a screen of patients at the Cripples' Home, Alton. This school was probably the best of its kind in the world. There children were kept in a position of rest for from eighteen months to two and a half years, and the results obtained were far in advance of what had been obtainable by surgical interference. The hygienic way of life was the most important factor in the open-air school. Rest, work, and nutrition conduced to a healthy life. The exercise should be organised, the child should be trained to personal cleanliness, and it should be suitably occupied. In an open-air school a child got more individual attention than in an ordinary school, and the same applied to medical attention. But it was idle to think that open-air schools offered a panacea for all children. There was the difficulty of classification to begin with, and he thought the classification should be based on physical and medical grounds. There were difficulties as to the home influences, where a child slept at home in a bad atmosphere for eight or nine hours a day. The conditions of success in the open-air school was that there should be adequate organisation and suitable and proper equipment; it was for what was called the pre-tuberculosis child that the open-air school had been designed. In conclusion, Sir George Newman spoke in very high terms of the work being carried on by Dr. Gauvain at the Alton School. Again was shown the need to make such schools wherever possible, and it is often not possible at first, residential institutions.

### RADIUM EMANATIONS IN COMMERCE.

The recent action of the Managers of the Radium Institute, referred to last week, in successfully prosecuting a private commercial enterprise which was trading under a name substantially similar, may usefully be considered in connection with a recent pronouncement by the Department of Agriculture of the United States. This Department, oddly enough, has jurisdiction under certain Foods and Drugs Acts, and is considering the question of prosecuting the vendors of radium emanations which are not of the potency they are represented to be. It appears that the public demand for radium emanations for any disease whatever has reached the dimensions of a craze; and the demand is largely met by a supply which is in large part fraudulent. The Department of Agriculture has a Bureau of Chemistry by which estimations of radio-activity of imported and other emanations are conducted. It is high time, in view of the commercialisation of radium generally we have mentioned, that some similar supervision should be undertaken here by the Board of Trade, or such other Government Department as possesses the requisite staff and powers.

### TRIBUTE TO A MEDICAL SUPERINTENDENT.

At a meeting of the public health and isolation hospital committee of the Acton Urban District Council a resolution was passed recording the committee's appreciation of the care displayed in the preparation and the general excellence of the annual report for 1912 of Dr. D. J. Thomas, medical officer of health and medical superintendent of the isolation hospital. This public testimony to the ability of Dr. Thomas must be gratifying to a hard-worked municipal official, as also to those who appreciate the good work he has accomplished in Acton, the capital of the laundry industry, where, in some 400 laundries, 4,000 hands find work, and not least of all because for some time a certain Acton councillor has been "ploughing a lonely furrow" in his endeavour to induce belief in a contention that since the erection of the Council's isolation hospital certain infectious diseases in the district have not decreased in number. Dr. Thomas, with fact and figure, has invariably contested such a claim, and this latest resolution of confidence is further evidence of the warm appreciation of his services by the Acton Urban District Council. The late Dr. Franklin Parsons has explained in a series of articles in THE HOSPITAL of late what is the true relation of hospital isolation to infectious disease.

### A TUBERCULOSIS DISPENSARY FOR SHOREDITCH.

Some twelve months ago there was started at the Royal Hospital for Diseases of the Chest in the City Road a department for the prevention of consumption. This was run on the lines of a tuberculosis dispensary, and much useful work was accomplished in Shoreditch, Finsbury, Islington, and Hackney, but principally in the first borough. Quite recently the Borough of Shoreditch has officially recognised this dispensary as their tuberculosis centre, and the work of the medical officer and the visiting sister will in future, as far as Shoreditch patients are concerned, be under the ægis of the Medical Officer of Health. This centre is also used to a considerable extent as a consultation department by the local medical practitioners, and two members of the staff of the Chest Hospital are available for additional expert advice. The medical officer in charge of the department from its commencement, Dr. H. Gibbins, has recently resigned, and has been succeeded by Dr. Lytton Maitland, M.D.Lond., D.P.H.Camb. The card-index system is employed freely in this department, and the smooth working of this method of keeping records is due to the large amount of labour expended by Dr. Gibbins in establishing the nicest order among the accumulated materials.

### NEW POSTS AT ST. JOHN'S HILL INFIRMARY.

The Wandsworth Board of Guardians, acting on the advice of Dr. Maccormac, their medical superintendent, have agreed to adopt a scheme for the better classification of the patients. They have decided that at St. James's Infirmary all cases requiring skilled nursing and active treatment shall be accommodated, whilst at St. John's Hill Infirmary those who do not require such a high standard of treatment shall be housed. Phthisis cases will be dealt with at St. James's Infirmary. Patients able in some degree to minister to their own needs will be accommodated at the Tooting Annexes, and male epileptic patients will be sent to Belmont. A reorganisation of the staff has been rendered necessary by these alterations. At St. James's Infirmary the nurses will be increased from 83 to 117, and it is proposed to appoint a third assistant medical officer. As far as possible attendants are to be substituted for nurses at St. John's Hill Infirmary; there the services of a junior assistant medical officer will be dispensed with, while at the Tooting Homes it is proposed to appoint about ten female attendants. When the scheme is in full working order it will greatly add to the better treatment of the sick and the more complete administration of the institutions.

### LONDON COUNTY COUNCIL AND LYING-IN HOMES

A month ago the London County Council decided to promote legislation to confer upon it powers in respect of the licensing and inspection of lying-in homes and institutions. In the report then submitted by the Midwives Act Committee it was stated that consideration had been given as to whether the regulation of these places could be best effected by means of registration or of licensing, and decided in favour of the latter. The attention of the Committee has now been directed to the fact that the Council, last week, when considering what means should be adopted for regulating nursing homes and massage establishments, decided to endeavour to obtain statutory powers of registration. The Committee has also been informed that the Secretary of State for the Home Department will oppose any application made by the London County Council for powers more stringent than registration in respect of lying-in homes. In these circumstances the London County Council proposes to proceed with the application to Parliament on the basis of registration instead of licensing.

### WOODEN HUTS FOR CONSUMPTIVES.

The Local Government Board has sent to local authorities an intimation that shelters for consumptives may be regarded as coming within the scope of dispensary treatment. So far as London is concerned some seven or eight Borough Councils are already providing huts on loan to patients who are likely to benefit by the open-air treatment. Usually these little wooden huts cost £12 each, and are so made that they can easily be erected and removed from a back garden. Last week, however, the Lambeth Council refused to buy any shelters for use in connection with its chief dispensary, but it was announced that such shelters were being provided by the authorities of St. Thomas's Hospital, who are, on behalf of the Council, providing the dispensary treatment for the northern wards of the borough. In a chest hospital or sanatorium such shelters can generally be made better and cheaper than those placed on the market. The price of £12 is, we think, an example of the latter's figure.

### SHOULD PHARMACISTS KNOW LATIN?

The Council of the Pharmaceutical Society has adopted a recommendation of the Parliamentary and General Purposes Committee to the effect that, among other matters, pharmaceutical students should not be required to submit evidence of having passed an examination in Latin. It advises Latin, however, to be chosen by candidates as one of the two optional subjects. Mr. E. White, the president, explained that, as Latin is now so rarely taught in primary and even in secondary schools, the above change in the compulsory part of the syllabus will admit by the Council the certificate of almost every boy who leaves a continuation or secondary school. Latin is still enforced by statute in the qualifying examination, so the effect of the alteration in the examination regulations appears to be to postpone the age at which evidence of Latin studies is required of the intending pharmacist. Mr. White also hinted that Latin, "but not academic Latin," would be incorporated in the prescription-reading part of the examination. "Not academic Latin" presumably means pharmacy Latin, which is at least better than none, just as Greek in the Oxford and Cambridge "Smalls" and "Little-go" can be defended as giving the candidate a knowledge of the Greek alphabet—a matter of immense value in any of the learned professions still.

### THIS WEEK'S DRUG MARKET.

Few quotable changes in the values of drugs have taken place during the week, but, as a consequence of the general dullness, there is a distinctly easier tendency in the prices of a number of products. It is somewhat difficult to account for the present quiet state of affairs, for at this season of the year the demand is, as a rule, fairly active. An improvement will doubtless set in shortly, and the downward tendency of prices will probably be checked. Among the drugs the prices of which are rather lower are oil of cloves, balsam tolu, menthol, and Japanese peppermint oil, and there is an easier tendency in the prices of cod-liver oil, castor oil, honey, and American peppermint oil. On the other hand, podophyllum root is somewhat dearer, and business in cascara sagrada has improved. At the recent public sale of drugs in Mincing Lane there was also a very poor demand; there were no buyers of Cape aloes, although lower prices would have been accepted; menthol sold at cheaper rates; asafetida was rather lower, as also was ipecacuanha.

